# Pre-mRNA splicing is a determinant of nucleosome organization

**DOI:** 10.1186/1756-8935-6-S1-P46

**Published:** 2013-03-18

**Authors:** Hadas Keren-Shaul, Galit Lev-Maor, Gil Ast

**Affiliations:** 1 Department of Human Molecular Genetics and Biochemistry, Sackler Faculty of Medicine, Tel Aviv University, Ramat Aviv 69978, Israel

## Background

Nearly 50% of human genetic diseases are an outcome of splicing errors and over 95% of our genes undergo alternative splicing [1,2]. Hence, understanding alternative splicing regulation is a key for understanding the complexity of the human genome. Alternative splicing is regulated by a variety of factors such as sequences in the pre-mRNA, splicing regulatory proteins, and transcription by RNA polymerase II. Recently, the organization of the chromatin has also been shown to affect alternative splicing [3,4]; however it is unknown whether alternative splicing can affect chromatin organization. Here we show for the first time that splicing, a process that occurs at the RNA level, can affect an upstream process at the DNA level – the organization of the chromatin.

## Materials and methods

We present a minigene system that exhibits a shift in splicing pattern in cells as a function of time following transfection, we evaluated whether the changes in splicing pattern were reflected by changes in nucleosome occupancy; we mutated the plasmid, used different drugs that affect chromatin structure, analyzed RNA polymerase II processivity. For all experiments MNase treatment and chromatin immunoprecipitation assay were used following QPCR analysis.

## Results

Chromatin organization affects alternative splicing and previous studies have shown that exons have increased nucleosome occupancy compared with their flanking introns [5]. To determine whether alternative splicing affects chromatin organization we developed a system in which the alternative splicing pattern switched from inclusion to skipping as a function of time. Changes in nucleosome occupancy were correlated with the change in the splicing pattern. Surprisingly, strengthening of the 5’splice site or strengthening the base pairing of U1 snRNA with an internal exon abrogated the skipping of the internal exons and also affected chromatin organization. Over-expression of splicing regulatory proteins also affected the splicing pattern and changed nucleosome occupancy. A specific splicing inhibitor was used to show that splicing impacts nucleosome organization endogenously [6,7]. The effect of splicing on the chromatin required a functional U1 snRNA base pairing with the 5’ splice site, but U1 pairing was not essential for U1 snRNA enhancement of transcription.

## Conclusions

Our data indicate that there is a delicate bi-directional interplay between chromatin organization and regulation of mRNA splicing. Regulation of alternative splicing at the RNA level influences an upstream process – the organization of the chromatin. U1 snRNA binding to the 5’ss provides the necessary signal from the splicing reaction back to chromatin organization.
